# Treatment of infertility and risk of breast cancer among women with a *BRCA* pathogenic variant: a matched case-control study

**DOI:** 10.1186/s12885-025-15146-0

**Published:** 2025-11-10

**Authors:** Marta Seca, Jacek Gronwald, Tomasz Huzarski, Karen Glass, Amber Aeilts, Raymond H. Kim, Beth Karlan, Christian F. Singer, Andrea Eisen, Nadine Tung, Olufunmilayo Olopade, Louise Bordeleau, Pal Moller, William D. Foulkes, Susan L. Neuhausen, Fergus Couch, Tuya Pal, Robert Fruscio, Cezary Cybulski, Jan Lubinski, Shana Kim, Ping Sun, Steven A. Narod, Joanne Kotsopoulos, Hereditary Breast Cancer Clinical Study Group, Tracy Graham, Kevin Sweet, Christine Elser, Georgia Wiesner, Aletta Poll, Ellen Warner, Larissa Peck, Emily Thain, Laura Redondo, Linda Steele, Howard Saal, Susan Neuhausen, Kim Serfas, Seema Panchal, Carey A. Cullinane, Robert E. Reilly, Daniel Rayson, Leanne Mercer, Jeffrey Dungan, Stephanie Cohen, Edmond Lemire, Stefania Zovato, Antonella Rastelli, Sophie Sun, Intan Schraeder, Leigha Senter, Joanne L. Blum

**Affiliations:** 1 Department of Medicine and Surgery, University of Milan-Bicocca, Piazza Dell’Ateneo Nuovo, Milan, Italy; 2 International Hereditary Cancer Center, Department of Genetics and Pathology, Pomeranian Medical University, Szczecin, Poland; 3 CReATe Fertility Centre, Toronto, ON Canada; 4 Department of Obstetrics and Gynecology, University of Toronto, Toronto, ON Canada; 5 Division of Human Genetics, the Ohio State University Medical Center, Comprehensive Cancer Center, Columbus, OH USA; 6 Division of Medical Oncology and Hematology, Princess Margaret Cancer Centre, University Health Network, University of Toronto, Toronto, ON Canada; 7 Department of Obstetrics and Gynecology, David Geffen School of Medicine, University of California, Los Angeles, Los Angeles, CA USA; 8 Department of Obstetrics and Gynecology and Comprehensive Cancer Center, Medical University of Vienna, Vienna, Austria; 9 Department of Medical Oncology, Sunnybrook Odette Cancer Center and University of Toronto, Toronto, ON Canada; 10 Department of Oncology, Juravinski Cancer Centre and McMaster University, Hamilton, ON Canada; 11 Beth Israel Deaconess Medical Center, Boston, MA USA; 12 Department of Medicine and Human Genetics, University of Chicago, Chicago, IL USA; 13 Department for Medical Genetics, The Norwegian Radium Hospital Oslo University Hospital, Oslo, Norway; 14 Department of Oncology, McGill Program in Cancer Genetics, McGill University, Montreal, QC Canada; 15 Department of Population Sciences, Beckman Research Institute of City of Hope, Duarte, CA USA; 16 Division of Experimental Pathology and Laboratory Medicine, Department of Laboratory Medicine and Pathology, Mayo Clinic, Rochester, MN USA; 17 Department of Medicine, Vanderbilt-Ingram Cancer Center, Vanderbilt University Medical Center, Nashville, TN USA; 18 UO Gynecology, Fondazione IRCCS San Gerardo, Via Pergolesi 33, Monza, Italy; 19 Women’s College Research Institute, Women’s College Hospital, 76 Grenville St., 6th Floor, Toronto, ON M5S 1B2 Canada; 20 Dalla Lana School of Public Health, University of Toronto, Toronto, ON Canada

**Keywords:** *BRCA1*, *BRCA2*, Infertility, IVF, Breast cancer

## Abstract

**Background:**

The global trend toward delayed childbearing has led to an increased use of fertility treatment, including in vitro fertilization (IVF) and hormonal medications. Concerns regarding the potential impact of these interventions on breast cancer risk, particularly among high-risk women with a pathogenic variant in the *BRCA1* or *BRCA2* genes remains an important clinical concern.

**Methods:**

We conducted a matched case–control analysis of women carrying a pathogenic or likely pathogenic variant in *BRCA1* or *BRCA2* enrolled in a longitudinal, international study. The analysis included 4,145 women with invasive breast cancer (cases) and 4,145 matched controls without breast cancer. Data on infertility and use of fertility treatments was collected by a research questionnaire. Conditional logistic regression was used to estimate the odds ratios (ORs) and 95% confidence intervals (CIs) for the association between infertility, fertility medications, and IVF, with the risk of breast cancer. Multivariable models were adjusted for parity and oral contraceptive use.

**Results:**

Among the 8,290 participants, 12% reported a history of infertility, 5% had used fertility medication, and 1% had undergone IVF. There was no statistically significant association between a history of infertility (OR = 0.96; 95% CI 0.84–1.10), use of any type of fertility medication (OR = 1.10; 95% CI 0.90–1.34), or IVF specifically (OR = 1.15; 95% CI 0.76–1.73) and the risk of *BRCA*-breast cancer. Findings were similar in the adjusted analyses.

**Conclusions:**

Findings from this large, international study found no evidence for an association between infertility or fertility treatment and the risk of breast cancer among *BRCA1* or *BRCA2* carriers. Although based on low rates of exposure, these findings provide some reassurance to *BRCA* carriers considering fertility treatment. Future studies evaluating impact of contemporary protocols are needed.

## Introduction

Concerns regarding the potential impact of the treatment of infertility on breast cancer risk persist, especially given the established etiologic links between reproductive factors (i.e., early menarche, late menopause) as well as hormonal factors (menopausal hormone therapy, oral contraceptives), and cancer risk [1]. Furthermore, in recent years, there has been a noticeable increase in the mean age at first birth, rising from 24.9 years in early 2000 to 30.3 years in 2021 [2]. This global trend has largely been driven by societal changes [3, 4] resulting in a concomitant increase in the use of artificial reproductive technology to enhance conception rates; in particular, in vitro* fertilization* (IVF), comprising intracytoplasmic sperm injection (ICSI), egg or embryo donation along with the use of several fertility drugs (i.e., clomiphene, letrozole, gonadotropins).

Women who inherit a pathogenic variant in the *BRCA1* or *BRCA2* gene (i.e., carriers) face complex family planning decisions given their heightened lifetime risks of developing breast and ovarian (or fallopian tube) cancer. Numerous studies have investigated whether treatment of fertility is a risk factor for breast cancer in the general population [5, 6] and the data has generally demonstrated no increased risk [7, 8]. There is less published on the topic specifically for *BRCA* carriers. It is plausible that fertility treatment may impact their breast cancer risk given that these regimens typically involve the use of rFSH and rLH that alter the normal ovarian cycle and create a transient hyper-estrogenic state [9]. Alternatively, the addition of aromatase inhibitors such as letrozole to more recent fertility protocols may confer protection.

Given the heightened risk of breast cancer among *BRCA* carriers, which includes a predilection to early-onset disease and development of more aggressive subtypes, it is of clinical importance to clarify whether a history of infertility per se or its treatment may be associated with an increased risk of *BRCA*-breast cancer. Thus, the goal of this study was to update our earlier report on the topic, including an additional 2,735 matched pairs, to assess whether a personal history of infertility is an independent risk factor for breast cancer, and furthermore, whether the use of fertility medication or IVF is associated with risk.

## Methods

### Study population

This study population has been previously described in detail [10]. Briefly, eligible participants were women with a confirmed pathogenic or likely pathogenic variant in the *BRCA1* and/or *BRCA2* gene (carriers) and who were enrolled in a longitudinal study with biennial data collection. This cohort was drawn from 85 individual participating centers from 17 countries. Germline mutation detection was performed using a variety of techniques, but all nucleotide sequences were confirmed using direct DNA sequencing. The study protocol was approved by the institutional ethics review board at Women’s College Hospital of all the participating centers and written informed consent was provided by each participant.

### Data collection

All participants completed a research questionnaire at the time of study enrollment (i.e., baseline) and a follow-up questionnaire every two years thereafter. Questionnaires were administered either in-person during a clinic appointment, or at a later date over the telephone or via mail/email. Both the baseline and follow-up questionnaires collect detailed information on known or suspected risk factors for breast and ovarian cancer, as well as information on personal and/or family history of cancer and other important factors including surgeries, reproductive and hormonal exposures, and medication use.

For the current study, we focused on information collected regarding self-reported history of infertility, as well as any treatments received. Specifically, information from the following questions was included: 1) ‘have you ever seen a doctor for a problem of difficulty in getting pregnant or in carrying a pregnancy, such as several miscarriages?’ (yes/no); and for those women who answered ‘yes’: ‘What reason did the doctor give to explain why you had trouble getting or staying pregnant?’; 2) ‘have you ever taken medication to increase your chances of becoming pregnant?’ (yes/no), and if the answer was ‘yes’: `name medication (s)’; how many months did you take this medication?’ ‘What years did you take this medication?’; and 3) ‘have you ever received fertility treatment such as in vitro fertilization (IVF)/embryo transfer to help you get pregnant?’ (yes/no). If ‘yes’: ‘what type of treatment did you receive?’.

Report of a fertility problem or medication was coded as ‘never or ever’. We also created three categories of fertility medication: 1) selective estrogen receptor modulators (SERMs) (i.e., clomiphene citrate, seraphine, SERM + recombinant FSH + LH); 2) gonadotropins (i.e., FSH, FSH + LH); and 3) progesterone (i.e., progesterone, dufaston); however, given the few number of women who used gonadotropins or progesterone, the latter two categories were combined in the analysis. Fertility treatment was coded as either: 1) IVF (i.e., IVF alone or IVF with embryo transfer) or 2) other non-IVF (i.e., intrauterine insemination, tubal surgery). Participants who did not indicate a fertility medication name or fertility type of treatment were classified as *‘missing’*.

### Inclusion and exclusion criteria

Women with a previous diagnosis of ovarian (*n* = 114*)* or other cancer (*n* = 189) and those who underwent oophorectomy prior to study enrollment (*n* = 280), or if they had tubal ligation prior to breast cancer (*n* = 1,846), were not considered eligible for inclusion in the current analysis. Participants were excluded if they had missing or incomplete information on personal history of breast (*n* = 95) or ovarian cancer (*n* = 182). Women were also excluded if they were missing information on oophorectomy status (*n* = 386), history of fertility treatment (*n* = 1,285) or were missing other important information (i.e., date of birth) (*n* = 10). After applying these exclusion criteria, there were 13,247 women potentially eligible for inclusion in the current study including 4,754 women with invasive breast cancer (potential cases) and 8,439 without a breast cancer diagnosis (potential controls).

### Statistical analysis

A matched case–control analysis was performed to evaluate the associations between a fertility problem (ever/never), use of fertility medication (ever/never) and fertility treatment (IVF or other) and the risk of *BRCA*-breast cancer. Cases were defined as women with a diagnosis of invasive breast cancer either at baseline or follow-up, while controls were women who had never had a diagnosis of breast cancer. Controls could not have had a preventive bilateral mastectomy prior to the date of diagnosis of the case. Cases and controls were matched on gene mutation (*BRCA1* or *BRCA2*), date of birth (within one year) and country of residence resulting in 4,145 matched pairs.

The student's *t*-test and the χ^2^ test were used to compare distributions of continuous and categorical variables between the cases and controls, respectively. Conditional logistic regression was used to estimate the odds ratio (OR) and 95% confidence intervals (CI) for breast cancer between the various infertility exposures and the risk of breast cancer, accounting for matching factors. Multivariate analysis further accounted for parity (one, two, three, four or more live births) and history of oral contraceptive use (ever/never). Use of fertility medication and fertility treatment was censored one calendar year prior to the breast cancer diagnosis of the matched case.

Analyses were conducted using SAS version 9.4 (SAS Institute, Cary, NC, USA). All tests were two-sided and were considered statistically significant if *P* < 0.05.

## Results

Table 1 summarizes the baseline characteristics of the 4,145 breast cases and 4,145 controls included in the current analysis. There was a total of 6,104 (74%) women with a *BRCA1* mutation and 2,186 (26%) women with a *BRCA2* mutation. Case and control subjects were similar with respect to age at menarche (13.1 vs 13.0 years; *P* = 0.07), oral contraceptive use (56.3% vs 57.7% *P* = 0.21) and mean parity (1.8 vs 1.8; *P* = 0.58); however, cases had a significantly early date of study enrollment compared to the controls (2006.8 vs. 2007.2; *P* = 0.007) and less likely to breastfeed (79% vs. 81%; *P* = 0.01)(Table 1). In the cases, 103 (2.5%) had an oophorectomy prior to the breast cancer diagnosis compared to 94 (2.3%) of the controls (*P* = 0.52).

**Table 1:** Characteristics of case and control subjects with a *BRCA1* or* BRCA2* mutation

Variables	Controls *n* = 4,145	Breast cancer cases *n* = 4,145	*P*^*a*^
Year of birth, mean (range)	1958.2 (1911–93)	1958.3 (1911–93)	0.99
Year at baseline, mean (range)	2007.2 (1993–22)	2006.8 (1993–22)	0.007
Age at diagnosis, mean (range)	n/a^b^	41.7 (19–77)	
*BRCA* mutation, *n* (%)			
*BRCA1*	3,052 (73.6)	3,052 (73.6)	
*BRCA2*	1,093 (26.4)	1,093 (26.4)	matched
Age at menarche, mean (range)	13.1 (8–20)	13.0 (8–18)	0.07
Parity, *n* (%)			
Never	665 (16.4%)	616 (15.2%)	
Ever	3,384 (83.6%)	3,427 (84.8%)	0.14
Mean	1.8 (0–8)	1.8 (0–8)	0.58
Missing	96	102	
Age at first birth, mean (range)^*c*^	25.2 (15–44)	25.3 (15–44)	0.66
Breastfeeding,* n* (%)^*c*^			
Never	543 (18.7)	630 (21.2)	
Ever	2,365 (81.3)	2,337 (78.8)	0.01
Mean	9.9 (0–147)	8.6 (0–130)	0.0001
Missing	476	460	
Oophorectomy, *n* (%)			
Never	4,051 (97.7)	4,042 (97.5)	
Ever	94 (2.3)	103 (2.5)	0.52
Oral contraceptive use, *n* (%)			
Never	1,788 (43.7)	1,735 (42.3)	
Ever	2,305 (56.3)	2,365 (57.7)	0.21
Missing	52	45	
Country of residence, *n* (%)^*d*^			
Canada	876 (21.1)	876 (21.1)	
Poland	1,519 (36.7)	1,519 (36.7)	
USA	1,346 (32.5)	1,346 (32.5)	
Other*	404 (9.8)	404 (9.8)	matched
BMI, mean (range)			
BMI at age 18	20.9 (11.6–44.3)	20.6 (10.2–43.9)	0.003
BMI at age 30	22.6 (13.1–43.9)	22.5 (12.5–44.4)	0.70
BMI at age 40	24.2 (13.5–44.9)	24.2 (12.5–44.5)	0.96
Fertility problem, n (%)			
Never	3,429 (86.9)	3,464 (87.4)	
Ever	515 (13.1)	502 (12.7)	0.59
Missing^*b*^	201	176	
Fertility medication, n (%)^*e*^			
Never	3,736 (94.8)	3,744 (98.2)	
Ever	204 (5.2)	224 (5.7)	0.36
Missing	205	177	
SERMs	79 (39)	77 (34)	
Gonadotropin/progesterone	49 (24)	52 (23)	
Missing	76 (37)	95 (43)	0.86
Duration of use (months)^*f*^	12.3 (0–144)	11.3 (0–120)	0.63
Fertility treatment, n (%)			
Never	4,080 (98.4)	4,070 (98.2)	
Ever, IVF	42 (1.0)	48 (1.2)	
Ever, Other non-IVF	23 (0.6)	27 (0.7)	0.69

^*^Austria, Italy, Netherlands, Israel, Norway, Sweden Bahamas, China, Latin America, Spain, United Kingdom

^a^All *P*-values are univariate and were derived using the Student’s *t*-test for continuous variables and the χ^2^ test for categorical variables. Missing data were excluded in the Student’s t-test and χ^2^ square

^b^n/a, not applicable

^c^Among parous

^d^Country of residence was that at the time of genetic testing

^e^SERMs: clomiphene citrate, serophene; gonadotropin: FSH or FSH/LH combination; progesterone: progesterone, dufaston

^f^Among women who reported use of a fertility drug

Among all the participants combined, 1,017 (12%) reported a fertility problem, 428 (5%) reported use of a fertility medication, and 90 (1%) received treatment for infertility (such as IVF, or other non-IVF treatment). There was no significant difference in these exposures by case or control status (*P* ≥ 0.36). The specific type of fertility medication used was available for 58% of the controls and 63% of the cases. Overall, SERMS were the most commonly used 79 (39%), followed by gonadotropin or progesterone-containing drugs 49 (24%). Proportions were similar among the cases and controls (*P* = 0.86) (Table 1).

There was no significant association between a history of infertility or use of fertility medication and the risk of breast cancer among women with a *BRCA1* or *BRCA2* mutation (Table 2). The univariate ORs were 0.96 (95% CI 0.84–1.10; *P* = 0.58) and 1.10 (95% CI 0.90–1.34 *P* = 0.35), respectively. These findings were similar in the multivariate model further adjusting for parity, breastfeeding and oral contraceptive use; the corresponding adjusted ORs were 0.95 (95% CI = 0.83–1.09, *P* = 0.48) and 1.09 (95%CI 0.89–1.33; *P* = 0.42). There was no significant association between receipt of IVF specifically (OR = 1.13; 95% CI 0.75–1.72;* P* = 0.56) or another non-IVF fertility treatment (e.g., IUI) (OR = 1.17, 95% CI 0.67–2.04; *P* = 0.58) (Table 2).

**Table 2:** Association between report of a fertility problem, use of a fertility medication, or IVF treatment and the risk of breast cancer in *BRCA1* and *BRCA2* mutation carriers

Variables	Univariate OR (95% CI)	*P*	Multivariate OR (95% CI)^a^	*P*
*Fertility problem*				
Never	1.00 (reference)		1.00 (reference)	
Ever	0.96 (0.84–1.10)	0.58	0.95 (0.83–1.09)	0.48
*Fertility medication*				
Never	1.00 (reference)		1.00 (reference)	
Ever	1.10 (0.90–1.34)	0.35	1.09 (0.89–1.33)	0.42
SERMS	0.97 (0.71–1.34)	0.88	0.97 (0.70–1.33)	0.85
Gonadotropin/progesterone	1.06 (0.72–1.57)	0.77	1.06 (0.71–1.57)	0.77
Missing	1.26 (0.92–1.71)	0.15	1.23 (0.90–1.68)	0.20
*Fertility treatment*				
Never	1.00 (reference)		1.00 (reference)	
IVF	1.15 (0.76–1.73)	0.52	1.13 (0.75–1.72)	0.56
Other	1.18 (0.68–2.05)	0.57	1.17 (0.67–2.04)	0.58

^a^ORs and 95% CI adjusted for parity (0,1,2,3, ≥ 4), breastfeeding history (ever/never) and oral contraceptive use (ever/never)

We also evaluated the association between the type of IVF medication and breast cancer risk (Table 2). There was no significant association between the use of a SERM or gonadotropin/progesterone-containing and breast cancer risk. The OR for use of a SERM was 0.97 (95%CI 0.70–1.33; *P* = 0.85) and was 1.06 (95%CI 0.71–1.57; *P* = 0.77) for the use of another type of drug.

Although based on small strata, findings were similar in the analysis stratified by *BRCA* mutation type or age at diagnosis < 50 vs. ≥ 50 (data not shown).

## Discussion

In this analysis of women with a *BRCA1* or *BRCA2* mutation, we explored whether there was an association between infertility per se, as well as the treatment of infertility, and the risk of developing breast cancer. Acknowledging the low rates of exposure overall, we found no significant association between history of infertility, use of a SERM or gonadotropin/progesterone-containing fertility medication, nor receipt of fertility treatment and risk. Although based on small strata, findings were similar in our analysis stratified by *BRCA* mutation and age at diagnosis. To our knowledge, this represents the largest study conducted to date and is an extension of our earlier report on the topic and includes an additional 2,735 matched pairs. The data generated align with findings for women at baseline population risk. Further studies are needed to evaluate more contemporary protocols, including the use of aromatase inhibitors, or among those undergoing preimplantation genetic testing.

To date, there have been two historical cohorts conducted specifically among *BRCA* carriers and findings are consistent with our current report [11, 12]. In the first publication on the topic, Derks-Smeets et al*.,* reported no significant relationship between ovarian stimulation for IVF and the risk of breast cancer (HR = 0.79 95% CI 0.46–1.36). The study included 2,514 *BRCA1* and *BRCA2* carriers and 938 incident cases with 3% of the population reporting a history of IVF treatment [12]. In the second analysis of 1,824 Jewish Israeli *BRCA* carriers and 687 incident cases, Perri et al*.,* similarly reported no association between treatment for infertility and breast cancer risk (HR = 0.65, 95% CI 0.39–1.08) [11]. These two prior studies were limited by the inclusion of a relatively young population with a low exposure rate and missing details on the specific type of fertility treatments received.

Liu et al*.* [13], recently summarized the data from eight reports of fertility treatment and breast cancer among women with a family history (defined as a history of breast cancer in at least one first- or second- or third-degree relative) (*n* = 5 studies) or a *BRCA* mutation (*n* = 3 studies including our previous report). They reported no significant association between receipt of any fertility treatment or the specific type of treatment (i.e., clomiphene citrate or gonadotropins) and breast cancer risk among women with a family history as well as those with a *BRCA* mutation.

Our null findings align with data from studies conducted among women at baseline population risk, generally reporting no relationship between infertility, the use of fertility medication and/or IVF and breast cancer risk [14]. In a recent meta-analysis of 25 studies (21 historical cohorts, two prospective and two case–control studies), Cullinane et al., published no association between with receipt of any fertility treatment and risk with (summary OR = 0.97, 95% CI 0.90–1.04) [7]. Findings were corroborated in a large prospective analysis of the Nurses’ Health Study which included 12,193 infertile women and 749 incident breast cancers after 30 years of follow-up [15]. Brinton et al*.,* reported in no relationship between a well-documented history of infertility and use of infertility drugs and risk of breast cancer. Findings from a large Danish population-based registry study of 96,782 infertile women and 1,234,070 fertile women, there were 20,567 incident cases after a median 20 years of follow-up. There was no association between fertility drugs and breast cancer risk among (HR = 1.02; 95% CI, 0.95–1.01) [16]. This association remained consistent even at subgroup analysis by drug type.

The significant role of sex hormones, particularly estrogens and progestins, in the development of breast cancer is widely acknowledged, and thus, it is plausible that infertility treatments may also impact breast carcinogenesis [5, 6, 8]. Nevertheless, delineating the potential association between IVF or fertility drug exposure and cancer risk is complex given the wide variation in treatment protocols. In the past, the “*long protocol*” was more commonly used; however, more recently, the “*antagonist protocol*” has become more widespread. Both protocols consist of follicular stimulation, and a trigger phase, while the *“long protocol”* also includes an initial ovarian suppression phase. All protocols are associated with a significant increase in circulating sex hormones, particularly estrogen, to stimulate the maturation of multiple eggs simultaneously [17].

It should be noted that drugs like clomiphene citrate were more commonly employed by women in our study [17]. Clomiphene acts as a selective estrogen receptor modulator (SERMs), similar to tamoxifen and thus may confer protection against breast cancer [18]. In our study, we found no such association, and the role of clomiphene remains contentious; some studies have suggested that high doses or multiple cycles of IVF with clomiphene might increase the risk of breast cancer [15, 19]. We also found no association between gonadotropin-containing fertility medications, which increase both circulating estrogen and progesterone levels, and risk. We did not evaluate the number of cycles received or the specific medications that were used.

In the last few years, there has been a rise in the use of controlled ovarian stimulation with aromatase inhibitors (i.e., letrozole) for fertility preservation, especially among women with a diagnosis of breast (or other) cancer prior to the initiation of chemotherapy [20]. Controlled ovarian stimulation with letrozole has been demonstrated to be safe, showing no difference in breast cancer recurrence rates. Notably, studies to date have demonstrated that letrozole is safe, with no increased risk of recurrence among breast cancer patients undergoing neoadjuvant or adjuvant chemotherapy. In fact, a recent systematic review and metanalysis of 15 studies on the topic reported a significantly lower recurrence rate in women who underwent fertility treatment compared to women not exposed to fertility treatment (RR = 0.58, 95% CI 0.46–0.73,* P* < 0.001) [21]. Unfortunately, even in our large dataset, there was only one report of letrozole use. It will be important to continue to follow these women given the burden of young cancer survivors who seek oncofertility treatment.

Our study is not without limitations. Even with our large dataset, we had low rates of exposure (~ 1% received treatment), and thus, we were not sufficiently powered to detect small to modest effect sizes. We relied on self-reported exposure (and outcome data) that was not confirmed by medical record review which may have introduced bias. Information regarding specific types of fertility medication was missing for a large proportion of participants and we did not include details on numbers of 46% of the cases and 34% of the controls, which may have resulted in misclassification and potential masking of an effect. We restricted to fertility issues that required medical consultation; however, it is plausible that women who experienced infertility did not seek medical care, resulting in underreporting. Finally, our study predominantly included participants from Canada, USA and Poland, restricting the generalizability of our findings. Despite these limitations, this remains the largest report on the topic specifically for *BRCA* carriers. Recall of infertility history obtained through self-administered questionnaires has previously been shown to be reliable [22] and our matched approach ensured cases and controls were similar for key characteristics and minimized impact of confounding.

Our findings provide some reassurance regarding the impact of fertility treatments on *BRCA*-associated breast cancer risk, aligning with data from studies conducted among women from the general population. With the increase in the use of fertility preservation for various reasons including an increasing age at first birth, oncofertility and for preimplantation genetic testing, it will be important to continue to report on cancer outcomes in this population who face the highest known risks of developing breast and ovarian cancer.

## Data Availability

Data will not be made available as the participants did not consent to sharing their data in future studies (or external collaborators). The datasets generated and/or analyzed during the current study are not publicly available but may be available from the corresponding author through a formal application and review process to the PI. If accepted, sharing may only be approved upon acceptable ethics and contract execution.

## References

[1] Narod SA. Modifiers of risk of hereditary breast cancer. Oncogene. 2006;25(43):5832–6. 16998497 10.1038/sj.onc.1209870

[2] Osterman MJK, Hamilton BE, Martin JA, Driscoll AK, Valenzuela CP. Births: final data for 2021. Natl Vital Stat Rep. 2023;72(1):1–53. 36723449

[3] Billari F, Frejka T, Hobcraft J, Macura M, van de Kaa D. Discussion of paper “explanations of the fertility crisis in modern societies: a search for commonalities”, Population Studies 57(3), 241–263, by John Caldwell and Thomas Schindlmayr. Popul Stud (Camb). 2004;58(1):77–92. 15204263 10.1080/0032472032000183762

[4] Frejka T, Sardon JP. A note on the cohort-fertility analysis in the paper “Patterns of low and lowest-low fertility in Europe”, Population Studies 58(2), 161–176, by Francesco C. Billari and Hans-Peter Kohler. Popul Stud (Camb). 2005;59(2):233–8. 16096200 10.1080/00324720500099645

[5] Travis RC, Key TJ. Oestrogen exposure and breast cancer risk. Breast Cancer Res. 2003;5(5):239–47. 12927032 10.1186/bcr628 PMC314432

[6] Key TJ, Pike MC. The role of oestrogens and progestagens in the epidemiology and prevention of breast cancer. Eur J Cancer Clin Oncol. 1988;24(1):29–43. 3276531 10.1016/0277-5379(88)90173-3

[7] Cullinane C, Gillan H, Geraghty J, Evoy D, Rothwell J, McCartan D, et al. Fertility treatment and breast-cancer incidence: meta-analysis. BJS Open. 2022;6(1). 10.1093/bjsopen/zrab149. PMID: 35143625; PMCID: PMC8830753. 10.1093/bjsopen/zrab149 PMC8830753 35143625

[8] Pike MC, Spicer DV, Dahmoush L, Press MF. Estrogens, progestogens, normal breast cell proliferation, and breast cancer risk. Epidemiol Rev. 1993;15(1):17–35. 8405201 10.1093/oxfordjournals.epirev.a036102

[9] Beckers NG, Laven JS, Eijkemans MJ, Fauser BC. Follicular and luteal phase characteristics following early cessation of gonadotrophin-releasing hormone agonist during ovarian stimulation for in-vitro fertilization. Hum Reprod. 2000;15(1):43–9. 10611186 10.1093/humrep/15.1.43

[10] Kotsopoulos J, Librach CL, Lubinski J, Gronwald J, Kim-Sing C, Ghadirian P, et al. Infertility, treatment of infertility, and the risk of breast cancer among women with BRCA1 and BRCA2 mutations: a case-control study. Cancer Causes Control. 2008;19(10):1111–9. 18509731 10.1007/s10552-008-9175-0

[11] Perri T, Naor-Revel S, Eliassi-Revivo P, Lifshitz D, Friedman E, Korach J. Fertility treatments and breast cancer risk in Jewish Israeli BRCA mutation carriers. Fertil Steril. 2021;116(2):538–45. 33823990 10.1016/j.fertnstert.2021.02.030

[12] Derks-Smeets IAP, Schrijver LH, de Die-Smulders CEM, Tjan-Heijnen VCG, van Golde RJT, Smits LJ, et al. Ovarian stimulation for IVF and risk of primary breast cancer in BRCA1/2 mutation carriers. Br J Cancer. 2018;119(3):357–63. 29937543 10.1038/s41416-018-0139-1 PMC6068188

[13] Liu X, Yue J, Pervaiz R, Zhang H, Wang L. Association between fertility treatments and breast cancer risk in women with a family history or BRCA mutations: a systematic review and meta-analysis. Front Endocrinol (Lausanne). 2022;13:986477. 36176466 10.3389/fendo.2022.986477 PMC9513064

[14] Klip H, Burger CW, Kenemans P, van Leeuwen FE. Cancer risk associated with subfertility and ovulation induction: a review. Cancer Causes Control. 2000;11(4):319–44. 10843444 10.1023/a:1008921211309

[15] Brinton LA, Scoccia B, Moghissi KS, Westhoff CL, Niwa S, Ruggieri D, et al. Long-term relationship of ovulation-stimulating drugs to breast cancer risk. Cancer Epidemiol Biomarkers Prev. 2014;23(4):584–93. 24700523 10.1158/1055-9965.EPI-13-0996 PMC3979528

[16] Guleria S, Kjær SK, Albieri V, Frederiksen K, Jensen A. A cohort study of breast cancer risk after 20 years of follow-up of women treated with fertility drugs. Cancer Epidemiol Biomarkers Prev. 2019;28(12):1986–92. 31533944 10.1158/1055-9965.EPI-19-0652

[17] Ovarian Stimulation TEGG, Bosch E, Broer S, Griesinger G, Grynberg M, Humaidan P, et al. ESHRE guideline: ovarian stimulation for IVF/ICSI. Hum Reprod Open. 2020;2020(2):hoaa009. 32395637 10.1093/hropen/hoaa009 PMC7203749

[18] Medicine PCotASfR. Use of clomiphene citrate in infertile women: a committee opinion. Fertil Steril. 2013;100(2):341–8. 23809505 10.1016/j.fertnstert.2013.05.033

[19] Gennari A, Costa M, Puntoni M, Paleari L, De Censi A, Sormani MP, et al. Breast cancer incidence after hormonal treatments for infertility: systematic review and meta-analysis of population-based studies. Breast Cancer Res Treat. 2015;150(2):405–13. 25744295 10.1007/s10549-015-3328-0

[20] Kim J, Turan V, Oktay K. Long-term safety of letrozole and gonadotropin stimulation for fertility preservation in women with breast cancer. J Clin Endocrinol Metab. 2016;101(4):1364–71. 26751194 10.1210/jc.2015-3878 PMC4880171

[21] Arecco L, Blondeaux E, Bruzzone M, Ceppi M, Latocca MM, Marrocco C, et al. Safety of fertility preservation techniques before and after anticancer treatments in young women with breast cancer: a systematic review and meta-analysis. Hum Reprod. 2022;37(5):954–68. 35220429 10.1093/humrep/deac035 PMC9071231

[22] Jung AM, Missmer SA, Cramer DW, Ginsburg ES, Terry KL, Vitonis AF, et al. Self-reported infertility diagnoses and treatment history approximately 20 years after fertility treatment initiation. Fertil Res Pract. 2021;7(1):7. 33712085 10.1186/s40738-021-00099-2 PMC7953690

